# Driving innovation in cancer symptom science through translational research: bridging science and practice

**DOI:** 10.1097/SPC.0000000000000792

**Published:** 2026-01-27

**Authors:** Juan J. Fierro, Seamus Coyle, Barry J.A. Laird, Lia van Zuylen

**Affiliations:** a Department of Medical Oncology, Amsterdam University Medical Center, Vrije Universiteit Amsterdam, Amsterdam, The Netherlands; b Cancer Center Amsterdam, Cancer Treatment and Quality of Life, Amsterdam University Medical Center, Amsterdam, The Netherlands; c Liverpool Head and Neck Cancer Centre, University of Liverpool, Liverpool, UK; d Department of Palliative Medicine, Clatterbridge Cancer Centre, Liverpool, UK; e Oslo University Hospital, University of Oslo, Oslo, Norway

**Keywords:** cachexia, delirium, palliative care, symptom prediction, translational research

## Abstract

**Purpose of review:**

Translational research is a dynamic process that aims to apply fundamental scientific discoveries into clinical practice through strong cooperation between scientists and healthcare providers. This review discusses recent advances in symptom science within palliative care, driven by translational research, and highlights the pressing need to bridge the gap between scientific innovation and clinical practice.

**Recent findings:**

The dramatic change in the cancer landscape in the last decade has been achieved through translational research. However, the role of translational research in symptom science in palliative care for patients with cancer has been chronically neglected. Recently, initial progress has been made in symptom prediction through biomarker discovery for distressing syndromes, such as delirium or cancer-related cachexia. Other areas where translational approaches offer promise include predicting survival and identifying the dying phase in patients with advanced cancer.

**Summary:**

Despite some progress in introducing translational research into palliative care, significant challenges persist, limiting the full potential of integrative and collaborative research to improve patient outcomes in cancer palliative care. Herein, key areas are discussed, including emerging themes and tools that could help bridge this gap.

## INTRODUCTION

Translational research acts as the bridge between developments in basic science and their implementation in clinical approaches, often described as ‘from bench to the bedside’ [1]. In recent years, the scientific community, biomedical research institutions, policymakers, and healthcare providers have shown increased interest in the role of translational research in different medical disciplines [2].

Translational research has led to significant achievements in oncology by unravelling the pathways implicated in disease development, progression, and treatment response. This has catapulted a wide variety of new therapeutics, such as immunotherapy and omics approaches for precision oncology, which have improved patient outcomes [3,4]. However, there is a paucity of data regarding translational research for improving symptoms in patients with advanced cancer, often the remit of palliative care specialists. This article seeks to examine recent advances in symptom science driven by translational research, particularly within palliative care, and to highlight their potential to transform the management of cancer-related symptoms.

**KEY POINTS**

- Translational research is a dynamic and multifaceted process that has led to breakthrough discoveries in oncology over the past decades; nevertheless, its role in palliative care remains unclear.
- There is a critical need to expand foundational research and accelerate its translation into clinical tools for cancer patients with end-stage disease undergoing palliative care.
- Translational research may be a powerful tool driving innovation in oncology palliative care
- Twenty-first-century biomedical technologies should be used to increase our understanding of symptom presentation and management in cancer.

## Translational research applied to symptom science in palliative care

Although research in palliative care has expanded over recent decades, studies in symptom science remain limited, despite the growing recognition of quality of life as a critical outcome for patients with cancer, sometimes considered as important as survival [5,6]. Contrary to what has been shown in oncology related to screening, diagnostics, and treatment, the translation process from basic science to improving patient care has been neglected in palliative care and research remains primarily clinical.

**FIGURE 1.**
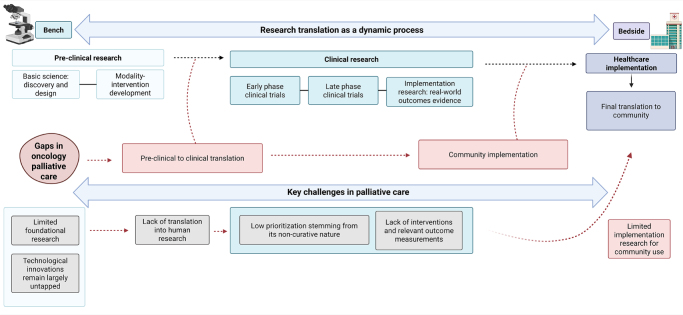
Translational research gaps in oncology palliative care. Research translation is a dynamic, bidirectional process, previously described as ‘from the bench to the bedside’. Nevertheless, key challenges remain unsolved for its implementation in palliative care.

Clear gaps in translational research within palliative care are depicted in Fig. 1. From this perspective, the main challenges to advancing translational research for better patient outcomes in palliative care include limited foundational research, inadequate application of contemporary experimental methods, insufficient translation of basic science into human studies, low prioritization of clinical testing, lack of designed interventions and relevant outcome measures, and limited implementation research for community use.

Notably, there are efforts to integrate translational research into palliative care, with a primary focus on symptom science. The following section outlines the most significant of these.

## Biomarkers for predicting and monitoring symptoms

Biomarkers are used for early diagnosis, prognosis, disease progression, and assessment of treatment response in patients with cancer [7]. Novel biomarkers hold promise in symptom science by helping predict when symptoms will occur and how patients will respond to treatment. Furthermore, biomarker research can deepen our understanding of the biological processes behind cancer-related symptoms, which are still not well understood. Interestingly, delirium, other neurological dysfunctions and cachexia–anorexia cancer-associated syndromes have benefited to a certain extent from the use of biomarkers as described below.

## The ongoing challenge of early delirium identification

Delirium is a neuropsychiatric condition and a frequent cause of acute cognitive impairment in patients with advanced cancer [8]. It has been linked to increased morbidity, mortality, and psychological distress of the patients and their caregivers [9,10]. Although early recognition and treatment are crucial for preventing adverse outcomes, achieving this in clinical practice remains challenging due to the heterogeneous nature of delirium symptomatology.

Clinical tools developed mainly for nurse bedside use, such as the Delirium Observation Screening scale, the 4 ‘A’s Test, the Confusion Assessment Method, and the Nursing Delirium Screening Scale, are used to assess patients at risk of developing delirium. However, these are mainly language-based tools, not consistently applied in clinical practice and require training and time to guarantee accurate assessment [11,12]. Despite these tools, there are reports of a high prevalence of underreported and underdiagnosed delirium among older hospitalized patients, resulting in undertreatment and poorer outcomes [13].

The pathogenesis and risk factors for developing delirium have been investigated with different clinical and translational methodologies, including neuroimaging, neurophysiological studies, and serum biomarkers. Delirium pathogenesis seems to involve multiple overlapping pathways, such as neuroinflammation, compromise of the blood–brain barrier, neuropathology, and neurodegeneration associated with ageing [14]. However, the sequence of events leading to the onset of delirium remains unclear, and the understanding of its biological etiology is limited.

Interleukin-6 (IL-6), IL-8, IL-10, C-reactive protein (CRP), and tumor necrosis factor-alpha (TNF-α) have been described as delirium biomarkers reflecting systemic and neuroinflammatory pathways [14–16^▪▪^]. In addition, neurofilament light chain, S100 calcium-binding protein B, and the neuron-specific enolase were studied as potential biomarkers reflecting neuronal damage [17–19]. However, they have been tested in heterogeneous scenarios, and none have been validated as diagnostic tools, impeding their use as sensitive instruments for the early identification of delirium.

New biomarkers with greater predictive power in the early stages of delirium could significantly improve clinical outcomes and care experience for patients with advanced cancer in hospital and outpatient settings. Further research is needed to standardize the biomarkers development methodology, expand current knowledge on delirium pathogenesis and translate it into pragmatic tools for use in clinical scenarios. To address this gap, translational approaches are needed, such as using animal models that mimic delirium-like states and conducting prospective studies in diverse patient populations (e.g., postoperative, critically or chronically ill individuals, with or without dementia). These studies should integrate clinical data with laboratory investigations of relevant disease pathways to help identify and develop targeted treatments. Additionally, advanced model analysis using machine-learning technologies shows promise for predicting delirium, though further research is required [20^▪^].

## Challenges in the prediction of neurological dysfunction resulting from cancer and its treatment

Cancer and its treatment may induce other central and peripheral neurological symptoms, such as cognitive impairment, chemotherapy-induced peripheral neuropathy or paraneoplastic neurological syndromes, affecting quality of life and treatment adherence [21]. These conditions are highly heterogeneous in clinical presentation, posing challenges for the development of reliable predictive models and standardized outcome measurements, which nowadays primarily rely on markers derived from functional diagnostic tools such as magnetic resonance imaging or electroencephalography. In recent years, interest in identifying novel biomarkers for early detection of neurological impairment and monitoring of treatment-related neurotoxicity has risen, leading to the introduction of promising biomarkers of neuroinflammation, including glial fibrillary acidic protein, as well as onconeural antibodies such as anti-Hu and anti-Ma for the detection of paraneoplastic neurological syndromes [22,23]. Nevertheless, most of these biomarkers remain in exploratory research, exhibit limited specificity, and are not yet standardized for clinical use. As a result, few reliable tools are available for the early identification of patients at risk of severe neurological impairment and for monitoring neuroimmune disruption across different cancer types. This represents an important yet understudied area within symptom science in oncology, underscoring the urgent need for concerted efforts to advance its translational development.

## Translational research impact through targeted treatment in cancer cachexia

Cachexia syndrome is characterized by weight loss (muscle ± fat) as a result of an underlying illness such as cancer, infection, or cardiovascular disease. Cachexia overall affects 33% of cancer patients, with prevalence rising to as high as 76% in advanced disease [24,25]. Cachexia is strongly associated with reduced overall survival and detrimental effects on quality of life, especially in advanced disease stages [25]. Previous efforts have aimed to unravel the underlying processes leading to cachexia in cancer patients, and promising tools are being translated into clinical practice. Currently, cachexia is considered a multi-organ syndrome that involves diverse pathological pathways such as chronic systemic inflammation, central-induced anorexia, gut dysbiosis, and fat metabolic disorder [26].

Systemic inflammation seems to play a pivotal role in cancer cachexia [27]. Increased TNF-α production leads to muscle wasting due to its catabolic effect through the E3 ligase pathway and the ubiquitin-proteosome system [28,29]. Chronic inflammation has been linked with anorexia induction through inhibition of hypothalamic neuropeptide Y and agouti gene-related protein neurons [30]. Additionally, transforming growth factor-beta signaling and resistance to ghrelin (a neuropeptide from gastrointestinal origin responsible for appetite stimulus and regulation of energy balance) contribute to an overall anorexic stimulus and subsequent weight loss in patients with cancer cachexia [31].

Translation of fundamental science discoveries concerning cancer cachexia pathophysiology allowed the proposal of new therapeutic schemes targeting the main implicated pathways. The use of ghrelin receptor agonists as an appetite stimulant has shown promising results in previous clinical trials, including improvements in lean and total body mass [32]. Moreover, previous studies have targeted the predominant systemic proinflammatory state using immunomodulatory agents such as thalidomide, which directly inhibit TNF-α production and NF-κB pathway activation [33]. This intervention has shown significant efficacy in attenuating weight loss and lean body mass depletion. However, its implementation is inconsistent due to the risk of adverse events, most pronounced when biomarkers of inflammation were used to stratify responders [34].

Attention has recently shifted toward the growth differentiation factor 15 (GDF-15), a stress-induced cytokine implicated in the anorexia and metabolic dysregulation characteristic of cancer cachexia. GDF-15 exerts its effects through the glial cell-derived neurotrophic factor receptor alpha-like (GFRAL–RET) complex in the hindbrain, promoting appetite suppression and energy expenditure. Elevated circulating GDF-15 levels correlate with weight loss, muscle wasting, and poor prognosis in cancer patients [35].

This has catalyzed the development of ponsegromab (PF-06946860), a humanized monoclonal antibody that neutralizes GDF-15. In animal models, GDF-15 inhibition reversed anorexia, reduced weight loss, and improved muscle function and survival [36]. Early-phase clinical data have provided compelling evidence that ponsegromab is both safe and effective: in a phase 2, randomized, placebo-controlled trial involving patients with non-small cell lung, pancreatic, or colorectal cancer and elevated GDF-15 levels, ponsegromab significantly increased body weight, appetite, physical activity, and muscle mass – particularly at the highest (400 mg) dose, which achieved weight gains exceeding 5% over 12 weeks [37]. Adverse events were comparable to placebo, and rates of nausea and vomiting were lower in ponsegromab-treated patients. Therefore, encouraging safety and efficacy findings have prompted the announcement of a pivotal phase 3 trial.

## Early and accurate risk estimation for timely palliative care: an unmet need

Early identification of patient needs based on symptom presentation and disease prognosis is crucial to guarantee that cancer patients receive appropriate care. By doing so, healthcare providers will be able to distinguish between patients with reversible symptom causes, those who would benefit from referral to specialized palliative care services, and those who require end-of-life care. Accurate screening and early palliative care involvement can have beneficial effects on quality of life and symptom intensity in cancer patients with advanced disease compared to standard care alone [38,39].

Recent advances in systemic cancer therapies, such as immunotherapy or combined regimes, and the enhanced recognition of tumor heterogeneity have improved patient outcomes but also introduced variability in disease trajectories, making prognostication increasingly difficult, especially when relying on traditional symptom-based or clinical models [40,41]. Moreover, the effect on survival of symptom burden and treatment-related toxicities cooperates with this. For instance, baseline high symptom burden consistently predicts poorer survival across cancer types and treatment settings [42]. In contrast, treatment-related toxicities may be associated with either improved or worsened survival depending on their severity and the patient’s individual characteristics [43,44]. These observations highlight the limitations of classic clinical prognostic models and underscore the need for translational research to elucidate the biological pathways underlying variability in disease trajectories across cancer types.

Along with early care needs recognition and accurate prognostication, identifying approaching death in patients with cancer is essential to adjust care and improve patients and their relatives’ experiences at the end-stage of the disease. However, healthcare providers usually encounter difficulties identifying patients with a short survival, starting the dying phase [45]. Several efforts strive to improve mortality prediction in advanced cancer. For instance, awareness of a systemic inflammatory response throughout different disease stages motivates the use of inflammatory biomarkers to predict death [46]. A prospective cohort study of end-stage cancer patients identified CRP, the CRP/albumin ratio, absolute neutrophil and lymphocyte counts, and the neutrophil-to-lymphocyte ratio (NLR) as markers with strong prognostic value and good discriminatory power for predicting death within the subsequent 90 days [47]. Moreover, a meta-analysis demonstrated the prognostic value of inflammatory markers such as CRP, albumin and NLR predicting survival in patients with advanced inoperable cancer across different tumor types and geographic locations [46].

Most studies predicting mortality in cancer patients continue to rely on conventional clinical observations, and the integration of emerging technologies – capable of identifying novel biomarkers with greater discriminatory power – remains overlooked for predicting mortality and identifying the dying phase in the context of advanced cancer. Interestingly, a metabolic analysis in urine from patients with lung cancer identified 125 metabolites that were differentially expressed in the last weeks of life. Moreover, a prediction model incorporating seven of these metabolites demonstrated discrimination power between patients at low, medium, or high risk of death within 30 days [48^▪^^▪^]. Similar initiatives employing omics technologies could help close the translational gap by offering essential insights into the biological pathways that lead to death, thereby improving the ability to predict outcomes.

## Seeking translation in symptom science

Translational research is a dynamic and multifaceted process that aims for close cooperation between fundamental scientists and healthcare providers in the pursuit of better interventions for patients [49]. Although there have been diverse recent advancements in oncology through translational research, we highlight that symptom science is a neglected area, and there is an urgent need for an integrated relationship between science and healthcare in patients with cancer receiving palliative support.

Ongoing initiatives targeting delirium and cachexia demonstrate how translational research could drive improvements in the management of distressing symptoms in patients with end-stage cancer. Nevertheless, the underlying biological mechanisms contributing to symptom presentation remain poorly understood and require further basic hypothesis-driven research. In addition, preclinical studies followed by clinical development testing and validation in real-world clinical scenarios are crucial for translating insights into therapeutic strategies with clinical value.

**FIGURE 2.**
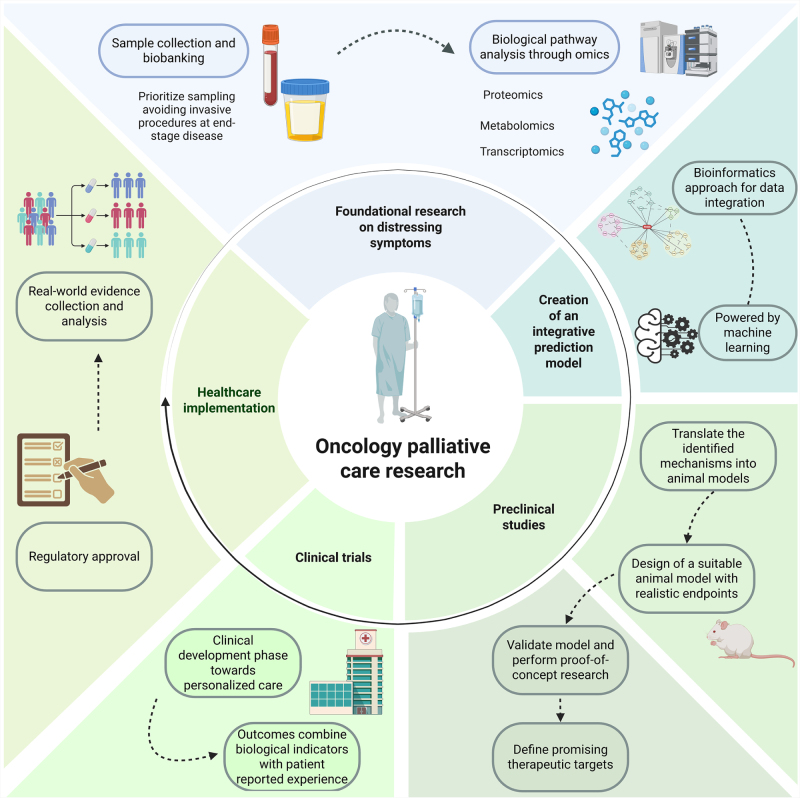
Implementation of translational research in oncology palliative care to drive personalized, patient-centered care.

Our proposal for implementing translational research in symptom science is illustrated in Fig. 2. As an initial step, a coordinated international effort is required to collect well-annotated samples, establishing a symptom-focused repository that can serve as a pipeline for targeted foundational research. Conducting basic research using cutting-edge technologies, integrating these discoveries into preclinical studies, and subsequently translating them into clinical trials that combine biological indicators with patient-reported experiences, is a significant challenge. However, elucidating the biological processes underlying distressing symptoms in cancer patients will pave the way towards better palliative care with novel therapeutics. Moreover, the application of recently available technologies, such as machine learning and artificial intelligence, could be crucial for accelerating progress toward improved patient outcomes.

It is acknowledged that modelling complex and fluctuating syndromes associated with advanced cancer, such as delirium and cachexia, is inherently challenging due to the heterogeneous interplay of risk factors, perpetuating factors, and individual differences in baseline vulnerability. Therefore, setting realistic and targeted objectives in the design of preclinical research in this field is advocated. For instance, while animal models hold substantial potential in advancing delirium research, we consider that they should prioritize the investigation of specific biological mechanisms relevant to the syndrome, rather than striving to reproduce its entire clinical phenotype.

Finally, engaging fundamental and clinical scientists, as well as increasing career development grant opportunities, can significantly advance the scientific frontiers of palliative care [50]. Funding opportunities prioritizing translational research for symptom management and end-of-life care, alongside curative approaches, are essential to reduce the burden of cancer. Equally important is the establishment of collaborative international networks that facilitate the sharing of data and biological samples, thereby accelerating discovery and improving the quality and generalizability of research findings.

## CONCLUSION

Translational research has achieved significant breakthroughs in cancer physiopathology and treatment; however, it has been barely implemented in symptom science. A notable gap exists between scientific innovation, technological development, and clinical scenarios in palliative care. Future efforts aimed at predicting and managing symptoms, supported by translational research tools, are crucial for improving patient care.

## References

[1] Fort DG Herr TM Shaw PL. Mapping the evolving definitions of translational research. J Clin Transl Sci 2017; 1:60–66. 28480056 10.1017/cts.2016.10 PMC5408839

[2] Acharjee A. Translational research and key aspects to make it successful. Transl Med Commun 2023; 8:1–3. 10.1186/s41231-023-00153-9 PMC11116195 38799298

[3] Basu A Budhraja A Abhilash D Gupta I. Novel omics technology driving translational research in precision oncology. Adv Genet 2021; 108:81–145. 34844717 10.1016/bs.adgen.2021.08.003

[4] Dragani TA Castells A Kulasingam V. Major milestones in translational oncology. BMC Med 2016; 14:110. 27469586 10.1186/s12916-016-0654-y PMC4964079

[5] Hong YR Huo J. Trends in U.S. population interest in palliative care and its association with prevalence of palliative care programs in U.S. states. J Pain Symptom Manage 2020; 60:e89–91. 32240753 10.1016/j.jpainsymman.2020.03.016

[6] Mehta AK Patel R Patel D Davis MP. Trends in published palliative care research: a 15-year review. Am J Hosp Palliat Med 2021; 38:489–493. 10.1177/1049909120944863 32705878

[7] Zhou Y Tao L Qiu J. Tumor biomarkers for diagnosis, prognosis and targeted therapy. Signal Transduct Target Ther 2024; 9:132. 38763973 10.1038/s41392-024-01823-2 PMC11102923

[8] Bush SH Lawlor PG Ryan K. Delirium in adult cancer patients: ESMO Clinical Practice Guidelines. Ann Oncol 2018; 29:iv143–65. 29992308 10.1093/annonc/mdy147

[9] Finucane AM Lugton J Kennedy C Spiller JA. The experiences of caregivers of patients with delirium, and their role in its management in palliative care settings: an integrative literature review. Psychooncology 2017; 26:291–300. 27132588 10.1002/pon.4140 PMC5363350

[10] Lawlor PG Bush SH. Delirium in patients with cancer: assessment, impact, mechanisms and management. Nat Rev Clin Oncol 2015; 12:77–92. 25178632 10.1038/nrclinonc.2014.147

[11] Park J Jeong E Lee J. The delirium observation screening scale: a systematic review and meta-analysis of diagnostic test accuracy. Clin Nurs Res 2021; 30:464–473. 33174438 10.1177/1054773820961234

[12] Lin CJ Su IC Huang SW. Delirium assessment tools among hospitalized older adults: a systematic review and metaanalysis of diagnostic accuracy. Ageing Res Rev 2023; 90:102025. 37527704 10.1016/j.arr.2023.102025

[13] Titlestad I Haugarvoll K Solvang SEH. Delirium is frequently underdiagnosed among older hospitalised patients despite available information in hospital medical records. Age Ageing 2024; 53:afae006. 38342753 10.1093/ageing/afae006 PMC10859244

[14] Smith CJ Hodge D Harrison FE, Roberson SW. The pathophysiology and biomarkers of delirium. Semin Neurol 2024;44:720–731. 10.1055/s-0044-1791666 PMC11622424 39419070

[15] Brummel NE Hughes CG McNeil JB. Systemic inflammation and delirium during critical illness. Intensive Care Med 2024; 50:687–696. 38647548 10.1007/s00134-024-07388-6 PMC12416785

[16.▪▪] Mosharaf MP Alam K Gow J Mahumud RA. Accumulating the key proteomic signatures associated with delirium: evidence from systematic review. PLoS One 2024; 19:e0309827. 39700095 10.1371/journal.pone.0309827 PMC11658594

[17] Kruize Z Van Campen I Vermunt L. Delirium pathophysiology in cancer: neurofilament light chain biomarker-narrative review. BMJ Support Palliat Care 2024; 15:319–325. 10.1136/spcare-2024-004781 PMC12128785 38290815

[18] Anderson BJ Reilly JP Shashaty MGS. Admission plasma levels of the neuronal injury marker neuron-specific enolase are associated with mortality and delirium in sepsis. J Crit Care 2016; 36:18–23. 27546742 10.1016/j.jcrc.2016.06.012 PMC5096992

[19] McNeil JB Hughes CG Girard T. Plasma biomarkers of inflammation, coagulation, and brain injury as predictors of delirium duration in older hospitalized patients. PLoS One 2019; 14:e0226412. 31856187 10.1371/journal.pone.0226412 PMC6922408

[20.▪] Kim YJ Lee H Woo HG. Machine learning-based model to predict delirium in patients with advanced cancer treated with palliative care: a multicenter, patient-based registry cohort. Sci Rep 2024; 14:11503. 38769382 10.1038/s41598-024-61627-w PMC11106243

[21] Mujinya R Owembabazi E Usman IM. Mechanisms of cancer-induced neurophysiological dysfunction and therapeutic strategies. Discov Oncol 2025; 16:1678. 40897950 10.1007/s12672-025-03520-0 PMC12405236

[22] Park Y Kc N Paneque A Cole PD. Tau, glial fibrillary acidic protein, and neurofilament light chain as brain protein biomarkers in cerebrospinal fluid and blood for diagnosis of neurobiological diseases. Int J Mol Sci 2024; 25:6295. 38928000 10.3390/ijms25126295 PMC11204270

[23] Zhang L Fan S Wang J. Antibody-positive paraneoplastic neurological syndromes associated with immune checkpoint inhibitors: a systematic review. J Neurol 2025;272:1–3. 10.1007/s00415-025-12992-7 40042691

[24] Sakaguchi T Maeda K Takeuchi T. Validity of the diagnostic criteria from the Asian Working Group for Cachexia in advanced cancer. J Cachexia Sarcopenia Muscle 2024; 15:370–379. 38115133 10.1002/jcsm.13408 PMC10834352

[25] Takaoka T Yaegashi A Watanabe D. Prevalence of and survival with cachexia among patients with cancer: a systematic review and meta-analysis. Adv Nutr 2024; 15:100282. 39127425 10.1016/j.advnut.2024.100282 PMC11402144

[26] Setiawan T Sari IN Wijaya YT. Cancer cachexia: molecular mechanisms and treatment strategies. J Hematol Oncol 2023; 16:54. 37217930 10.1186/s13045-023-01454-0 PMC10204324

[27] Balsano R Kruize Z Lunardi M. Transforming growth factor-beta signaling in cancer-induced cachexia: from molecular pathways to the clinics. Cells 2022; 11:2671. 36078078 10.3390/cells11172671 PMC9454487

[28] Patel HJ Patel BM. TNF-α and cancer cachexia: molecular insights and clinical implications. Life Sci 2017; 170:56–63. 27919820 10.1016/j.lfs.2016.11.033

[29] Yuan L Han J Meng Q. Muscle-specific E3 ubiquitin ligases are involved in muscle atrophy of cancer cachexia: an in vitro and in vivo study. Oncol Rep 2015; 33:2261–2268. 25760630 10.3892/or.2015.3845

[30] Mendes MCS Pimentel GD Costa FO Carvalheira JBC. Molecular and neuroendocrine mechanisms of cancer cachexia. J Endocrinol 2015; 226:R29–43. 26112046 10.1530/JOE-15-0170

[31] McGovern J Dolan RD Skipworth RJ. Cancer cachexia: a nutritional or a systemic inflammatory syndrome? Br J Cancer 2022; 127:379–382. 35523879 10.1038/s41416-022-01826-2 PMC9073809

[32] Dev R Amano K Naito T Del Fabbro E. Anamorelin for the treatment of cancer anorexia-cachexia syndrome. Curr Oncol Rep 2024; 26:762–772. 38771469 10.1007/s11912-024-01549-y

[33] Keifer JA Guttridge DC Ashburner BP Baldwin AS. Inhibition of NF-κB activity by thalidomide through suppression of IκB kinase activity. J Biol Chem 2001; 276:22382–22387. 11297551 10.1074/jbc.M100938200

[34] Reid J Mills M Cantwell M. Thalidomide for managing cancer cachexia. Cochrane Database Syst Rev 2012; 2012:CD008664. 10.1002/14651858.CD008664.pub2 PMC6353113 22513961

[35] Breen DM Kim H Bennett D. GDF-15 neutralization alleviates platinum-based chemotherapy-induced emesis, anorexia, and weight loss in mice and nonhuman primates. Cell Metab 2020; 32:938–950.e6. 33207247 10.1016/j.cmet.2020.10.023

[36] Kim-Muller JY Song LJ LaCarubba Paulhus B. GDF15 neutralization restores muscle function and physical performance in a mouse model of cancer cachexia. Cell Rep 2023; 42:111947. 36640326 10.1016/j.celrep.2022.111947

[37] Groarke JD Crawford J Collins SM. Ponsegromab for the Treatment of Cancer Cachexia. N Engl J Med 2024; 391:2291–2303. 39282907 10.1056/NEJMoa2409515

[38] Haun MW Estel S Rücker G. Early palliative care for adults with advanced cancer. Cochrane Database Syst Rev 2017; 2017:CD011129. 10.1002/14651858.CD011129.pub2 PMC6481832 28603881

[39] Strang P. Palliative oncology and palliative care. Mol Oncol 2022; 16:3399–3409. 35762045 10.1002/1878-0261.13278 PMC9533690

[40] Zhang M Liu C Tu J. Advances in cancer immunotherapy: historical perspectives, current developments, and future directions. Mol Cancer 2025; 24:136. 40336045 10.1186/s12943-025-02305-x PMC12057291

[41] Shen H Zheng Q Pan J. Intra-tumor heterogeneity-resistant gene signature predicts prognosis and immune infiltration in breast cancer. Front Immunol 2025; 16:1598858. 41080607 10.3389/fimmu.2025.1598858 PMC12511067

[42] Batra A Yang L Boyne DJ. Associations between baseline symptom burden as assessed by patient-reported outcomes and overall survival of patients with metastatic cancer. Support Care Cancer 2021; 29:1423–1431. 32676854 10.1007/s00520-020-05623-6

[43] Othus M Patel SP Chae YK. First cycle toxicity and survival in patients with rare cancers treated with checkpoint inhibitors. J Natl Cancer Inst 2025; 117:692–700. 39565908 10.1093/jnci/djae297 PMC11972677

[44] Pinato DJ Marron TU Mishra-Kalyani PS. Treatment-related toxicity and improved outcome from immunotherapy in hepatocellular cancer: evidence from an FDA pooled analysis of landmark clinical trials with validation from routine practice. Eur J Cancer 2021; 157:140–152. 34508996 10.1016/j.ejca.2021.08.020

[45] Hui D. Prognostication of survival in patients with advanced cancer: predicting the unpredictable? Cancer Control 2015; 22:489–497. 26678976 10.1177/107327481502200415 PMC4769860

[46] Dolan RD McSorley ST Horgan PG. The role of the systemic inflammatory response in predicting outcomes in patients with advanced inoperable cancer: systematic review and meta-analysis. Crit Rev Oncol Hematol 2017; 116:134–146. 28693795 10.1016/j.critrevonc.2017.06.002

[47] Cunha G da C Rosa KS da C Wiegert EVM de Oliveira LC. Clinical relevance and prognostic value of inflammatory biomarkers: a prospective study in terminal cancer patients receiving palliative care. J Pain Symptom Manage 2021; 62:978–986. 33895281 10.1016/j.jpainsymman.2021.04.009

[48.▪▪] Coyle S Chapman E Hughes DM. Urinary metabolite model to predict the dying process in lung cancer patients. Commun Med 2025;5:49. 40016594 10.1038/s43856-025-00764-3 PMC11868640

[49] Fernandez-Moure JS. Lost in translation: the gap in scientific advancements and clinical application. Front Bioeng Biotechnol 2016; 4:202918. 10.3389/fbioe.2016.00043 PMC4891347 27376058

[50] Schenker Y Ellington L Bell L. The national postdoctoral palliative care research training collaborative: history, activities, challenges, and future goals. J Palliat Med 2021; 24:545–553. 32955969 10.1089/jpm.2020.0411 PMC8182655

